# Cardiac vagal modulation predicts decision-making in sacrificial and everyday moral dilemmas

**DOI:** 10.1038/s41598-025-96475-9

**Published:** 2025-04-15

**Authors:** Rebecca Prell, Martina Anna Maggioni, Katrin Starcke

**Affiliations:** 1 University of Duisburg-Essen, General Psychology: Cognition, Forsthausweg 2, 47057 Duisburg, Germany; 2 Berlin Fire Brigade, Research Department, Berlin, Germany; 3 Charité—Universitätsmedizin Berlin, Institute of Physiology, Center for Space Medicine and Extreme Environments Berlin, Berlin, Germany; 4 Department of Biomedical Sciences for Health, Università degli Studi di Milano, Milan, Italy; 5 SRH Berlin University of Applied Sciences, Berlin, Germany; 6 Berlin Institute of Biomusicology and Empirical Research, Berlin, Germany

**Keywords:** Heart rate variability (HRV), Self-regulation, Moral decision-making, Sacrificial dilemmas, Everyday moral dilemmas, Physiology, Psychology, Biomarkers, Cardiology

## Abstract

Cardiac vagal modulation indicates the self-regulatory activity of the autonomic nervous system and can thus be used to assess an individual’s ability to integrate physiological, cognitive, and emotional responses in the decision-making process. In this paper, cardiac vagal modulation, quantified by heart rate variability (HRV), was used to predict decision-making in two types of moral dilemmas: First, sacrificial dilemmas which create a moral conflict between utilitarian (acting to maximise aggregate well-being by sacrifice) and deontological (rejecting the sacrifice) alternatives; second, everyday dilemmas which create a conflict between altruistic (acting non-selfish) and egoistic (acting self-centered) alternatives. HRV was analysed in 112 healthy participants before, during, and after decision-making, allowing to assess not only self-regulatory ability (resting HRV) but also self-regulatory capacities (HRV during and after decision-making). HRV predicted concrete decisions, but effects were only found in women. In sacrificial dilemmas, deontological women showed a stronger vagal withdrawal but also a faster cardiac vagal recovery compared to high utilitarian women. In everyday moral dilemmas, altruism was associated with reduced vagal outflow and increased sympathetic activity. Findings suggest differences in the acquisition of self-regulatory capacities between decision-makers in both types of moral dilemmas, which can be measured by HRV.

**Supplementary Information:**

The online version contains supplementary material available at 10.1038/s41598-025-96475-9.

## Introduction

Moral decisions pose intellectual and emotional challenges for humankind. Probably the best-known example is the classic trolley dilemma^1^, in which an out-of-control trolley is about to run over five workers on the track ahead. The only way to save them is to pull a lever, sending the trolley onto a side track, but this would kill another worker standing on that siding. Decisions in such dilemmas supposedly reflect two philosophical currents: utilitarianism^2^ and deontology^3^. Utilitarianism holds that decisions are desirable if they bring the greatest benefit to the greatest number of people (e.g., pulling the lever to sacrifice one person for the benefit of the other five). Deontology holds that decisions are based on one’s own moral convictions (i.e., refusing to harm one person even if it benefits more people). The dual process theory^4,5^ claims that the deontological decision is grounded in aversive emotional reactions to harming a person, while (cognitive) self-regulation in the form of laborious moral deliberation is required to overcome the prepotent deontological emotion and make the decision to harm a person to achieve a higher-order utilitarian goal. Supporting this assumption, cognitive and emotional load interfere with utilitarian decision-making. Studies have shown that acute stress^6–8^, chronic stress^9,10^, or difficulties in emotion regulation^11^ lead to fewer utilitarian decisions in sacrificial dilemmas such as the trolley dilemma.

While moral decision-making in hypothetical extreme situations is fairly well-researched, studies on everyday moral dilemmas are underrepresented. Those require a decision between two conflicting needs: forgoing personal profit for the benefit of the collective (altruistic option) versus fulfilling personally oriented benefits (egoistic option). Similar to the sacrificial dilemmas, a person’s self-regulation is linked to the concrete decision. In a recent neuroimaging study, the anterior cingulate cortex—a brain structure involved in empathy for pain—was active in people who made altruistic decisions in a Pain versus Gain task (financial self-benefiting decision versus another person’s pain), suggesting that empathic concern requires self-regulation, which ultimately induces altruistic motivation^12^.

In sum, moral inclinations appear to be based on processes that require self-regulation of cognitive and emotional responses in the decision-making process. One promising tool that appears well-suited to understanding how people draw on cognitive or emotional self-regulation when making (moral) decisions is heart rate variability (HRV). The HRV reflects the time variations between successive heartbeats, which are regulated by the autonomic nervous system through excitatory inputs from the sympathetic nervous system and inhibitory inputs from the vagus nerve^13^. According to the neurovisceral integration model^14, the^ brain regions—ventromedial prefrontal cortex/vmPFC, amygdala, hypothalamus—that are crucial for decision-making are bidirectionally connected to the sympathetic and vagal branches of the autonomic nervous system, which modulate cardiac autonomic activity^15,16^. Thus, HRV provides information about the functioning of these circuits and the self-regulatory ability as well as capacity in decision-making. Given this nexus, HRV analysis could be used to objectively assess how people’s moral decision-making is predicted by self-regulation. The present study aims to shed light on this relationship. Results could be used for targeted interventions, particularly in stressful decision situations. The implications are far-reaching, expanding our understanding of the brain-body connection for decision-making.

### Moral decision-making and HRV

Research suggests that better self-regulation ability can be indicated by effective activation of the vagus nerve, which can be measured by vagally mediated (vm) resting HRV. This nexus is fairly well supported by meta-analytic research linking lower resting vmHRV to stress^16^, several self-regulatory difficulties such as poorer top-down self-regulation (e.g., executive function, emotion regulation, effortful control)^17^, and poorer performance in cognitive domains^18^. On this basis, it is widely postulated that a higher resting vmHRV is associated with greater physiological, emotional, and cognitive resources available for self-regulation.

As utilitarian decision-making appears to be rooted primarily in deliberation (see dual process theory^4,5^), and higher resting vmHRV has been associated with greater self-regulation ability^14,15^, it can be argued that *high* resting vmHRV would predict utilitarian decision-making. However, the empirical basis for such a claim is sparse and contradictory. In an exploratory approach, Fooken and Schaffner^19^ used a single sacrificial dilemma in which participants could only save one person’s life at the cost of another’s. During the decision-making, emotional stress levels were assessed using an HRV index of sympatho-vagal balance (LF/HF ratio). No relationship between the concrete decision and HRV cloud be demonstrated. Park et al.^20^ used a series of sacrificial dilemmas^21^ and found that *low* resting vmHRV correlated with high utilitarian tendencies. They interpret that low resting vmHRV indicates a poor ability to integrate visceral responses, moderating moral decision-making. These results could be replicated by Rosas et al.^22^ who used sensitivity to utilitarian gradients in a set of five sacrificial dilemmas as a measure of moral performance. The utilitarian responses were negatively correlated with high resting HRV parameters. Another study could not replicate the original findings by Park et al.^20^, although after controlling for sex and hormonal contraceptives, HRV was positively correlated with deontological decisions in women and men^23^.

In sum, incorporating the dual process theory with the neurovisceral integration model, a high resting HRV, representing effective self-regulatory ability, should be related to utilitarian decisions. However, the results of the above-mentioned physio-psychological studies show the contrary: A high vmHRV is maladaptive for utilitarian decision-making. Possible explanations arise from the literature. For example, a recent study showed that a high vmHRV was *positively*,* rather than negatively* associated with higher switching costs in an emotion appraisal flexibility task, indicating maladaptive emotional flexibility^24^. Neuroimaging data revealed that people with self-regulatory impairment due to damage to the vmPFC are more likely to make utilitarian moral judgments^25^ and to approve personal moral violations^26,27^. Also, utilitarian decision-making has been associated with antisocial traits, such as primary psychopathy or alexithymia (emotional blindness)^28^. This gap between theory and empirical findings is the nexus of this study.

### Current study

The scope of this study is to investigate self-regulation via HRV in different moral decision-making situations. This contributes to a better understanding of the integration of self-regulatory processes into moral decision-making. Advancing the debate, this study considers key methodological facets: first, expanding the singular view of sacrificial dilemmas by including everyday moral dilemmas, second, considering multiple HRV measurement times, and third, considering sex-specific differences in the analysis.

First, the focus is broadened to everyday moral dilemmas because these dilemmas are much more common in ‘normal life’ and should elicit weaker emotional responses than sacrificial dilemmas^29^. This investigation is particularly valuable because there is a lack of understanding of how sensitive HRV is in detecting self-regulatory efforts in everyday decision-making, as these dilemmas have been insufficiently considered to date. Second, this study specifically looks at different HRV measurement times (i.e., HRV at rest, during decision-making and recovery). To date, research has focused on resting HRV, and therefore, no conclusions can be drawn about stimulus-induced changes and self-regulatory dynamics in moral decision-making. Third, recent studies suggest that there are HRV differences between women and men, such that women have a higher mean heart rate but also a higher HRV^30–32^. Especially when investigating moral decision-making, studies have either relied on mixed-sex samples without considering, for example, hormonal differences that influence HRV^22^, or statistically controlled for sex without examining possible interaction effects^20^. This is particularly noteworthy, as the latest replication study in this field clearly demonstrates sex effects^23^. Inadequate attention to sex in HRV studies is problematic because potential effects remain undetected, or results are incorrectly assumed to be universal for both sexes, ultimately limiting the generalisability and applicability of the results. To address this issue, the present study specifically accounts for sex differences by stratifying the sample, ensuring a more valid interpretation of the results and gaining more precise insights into the relationships between emotional processing in moral decision-making and HRV.

In sum, this study pursues two main objectives: First, to examine the self-regulatory demands of moral dilemma tasks, and second, to employ HRV analysis as a tool to differentiate between concrete moral decisions in sacrificial and everyday moral dilemmas to identify different patterns in the utilisation of self-regulatory capacities.

*Sacrificial Moral Decision-Making*: Regarding the self-regulatory demands of the sacrificial moral dilemmas, vmHRV is expected to show a pattern resembling a replenishment effect: compared to rest, vagal tone increases during decision-making in sacrificial dilemmas and then gradually decreases during recovery. Increased cardiac vagal control during decision-making would be equivalent to adaptive self-regulation (e.g., in terms of enhanced concentration)^33^. Regarding the different patterns in the utilisation of self-regulatory capacities, low vmHRV may predict fewer utilitarian judgments, as the decision to harm a person to achieve a higher-order utilitarian goal seems to be the result of self-regulation in the form of moral deliberation, emotional regulation, or cognitive effort. Therefore, although there is evidence to the contrary as described above, low utilitarian decision-makers are expected to have a greater sympathetic response and vagal withdrawal during decision-making due to aversive emotional responses. As a result of this greater utilisation of regulatory capacity, slower HRV recovery is expected in low utilitarians.

*Everyday moral Decision-Making*: Regarding the self-regulatory demands of everyday moral dilemmas, a replenishment effect is expected, similar to sacrificial dilemmas, which could be interpreted as adaptive self-regulation. Regarding the different patterns in the utilisation of self-regulatory capacities, low vmHRV may predict fewer altruistic judgements, as the activation of the vagus nerve is discussed as a driver of prosocial behaviour or social engagement (polyvagal theory^34^). Also, altruistic feelings (e.g., experience of compassion, empathetic concern) are associated with an increase in vagal activity^35,36^. This has not yet been tested in everyday moral dilemmas, but an increase in vagal activity during decision-making may reflect self-regulatory resources/efforts aimed at accepting personal discomfort for the benefit of others. Therefore, low altruistic decision-makers are expected to have a stronger sympathetic response during decision-making and subsequently a slower HRV recovery.

## Results

The final sample consisted of 112 individuals (77 women). Women had a mean age of 25.7 years (SD = 5.65), and men of 28.2 years (SD = 4.35).

### Sacrificial moral decision-making

**Fig. 1:**
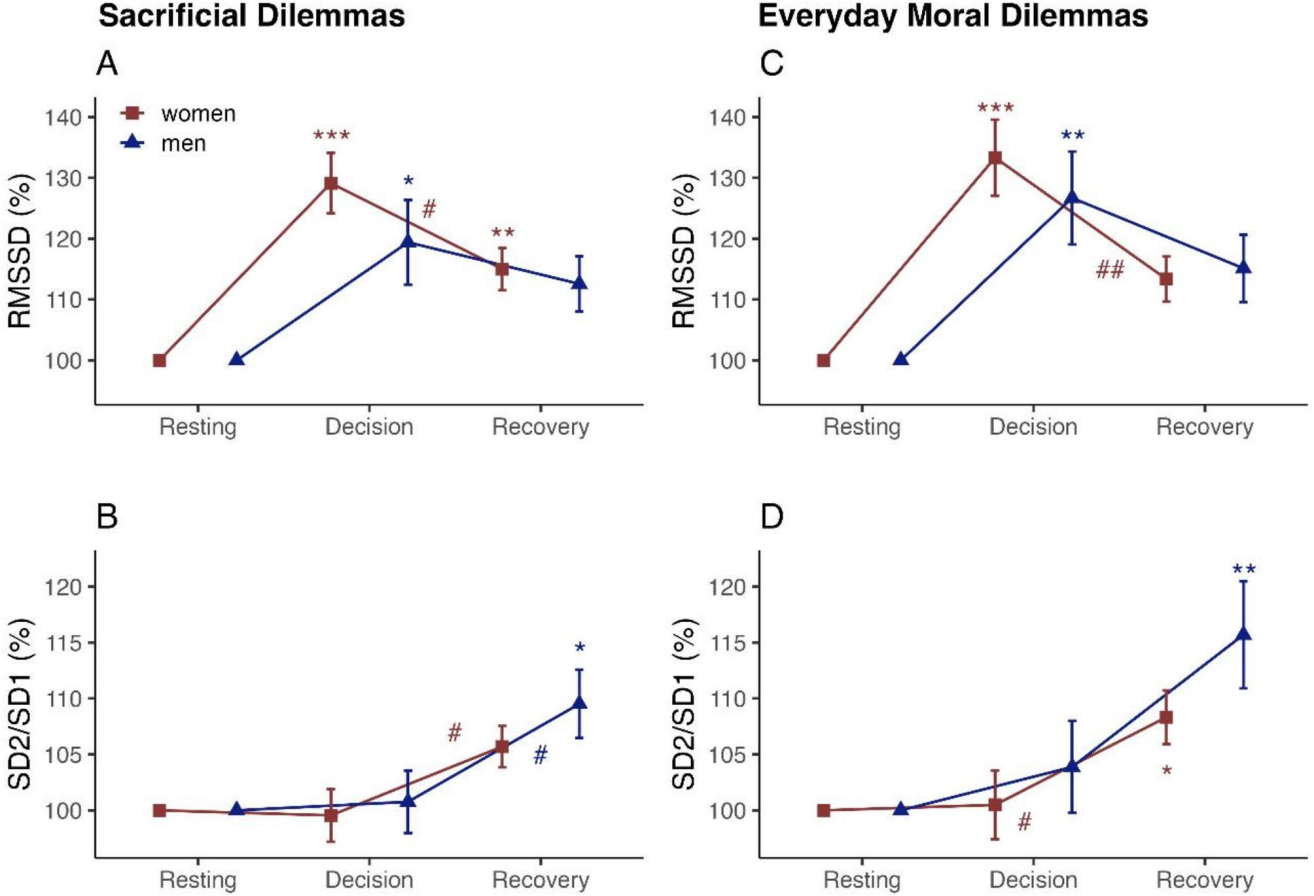
Self-regulatory demands of decision-making as HRV changes across measurements. HRV indices in women (red squares) and men (blue triangles). Left: sacrificial moral dilemmas. Right: everyday moral dilemmas. Measurement times: before (Resting), during decision-making (Decision) and afterwards (Recovery). Data are presented for decision-making and recovery as mean ± SE of percentage change from resting (i.e., 100% reference category). Coloured asterisks (*) indicate significance compared to resting within the group. Coloured hashes (#) indicate significance between decision-making and recovery within the group. (**A** and **C**) RMSSD, root mean square of successive differences; (**B** and **D**) SD1/SD2, short-term to long-term HRV. *** *p* < 0.001, **## *p* < 0.01, *# *p* < 0.05.

The first study objective related to the self-regulatory demands of sacrificial moral decision-making, for which HRV was analysed using repeated measures ANOVA with Tukey’s HSD test for multiple comparisons across the three measurement times: resting, decision-making, and recovery. In both women and men, cardiac vagal tone increased during sacrificial decision-making compared to rest (women’s RMSSD by 29.11%, *p* < 0.001; men’s RMSSD by 19.40%, *p* < 0.05). In women, cardiac vagal tone decreased during recovery (RMSSD by -14.11%, *p <* 0.05), so the difference between resting and recovery was 14.99% (*p* < 0.01). Men had a smaller vagal decrease during recovery (RMSSD by -6.84%, *p* = 0.57) and subsequently no significant difference between resting and recovery (*p* = 0.15). Yet, these differences between women and men were not significant (*p* = 0.64). Complementary, DFA1 increased by 6.41% in women (*p* < 0.05) but only slightly by 3.38% (*p* = 0.48) in men during decision-making. While DFA1 stagnated in women during recovery (-0.39%, *p* = 0.98), it increased in men (7.35%, *p* < 0.05), ending in a significant difference between resting and recovery (10.74%, *p* < 0.01) for men, but narrowly not for women (6.02%, *p* = 0.06). Yet, this difference between women and men was also not significant (*p* = 0.66). Starting from the resting phase, through decision-making to recovery, the sympatho-vagal balance increased in both women and men throughout the measurements (women’s LF/HF by 44.60, *p* < 0.01; men’s LF/HF by 71.79, *p* < 0.01). Figure 1, panels A and B, shows percentage changes from resting to sacrificial moral decision-making and recovery in selected HRV indices. Detailed results for all HRV indices are summarised in the Supplementary Material (Table S1-S2).

**Fig. 2:**
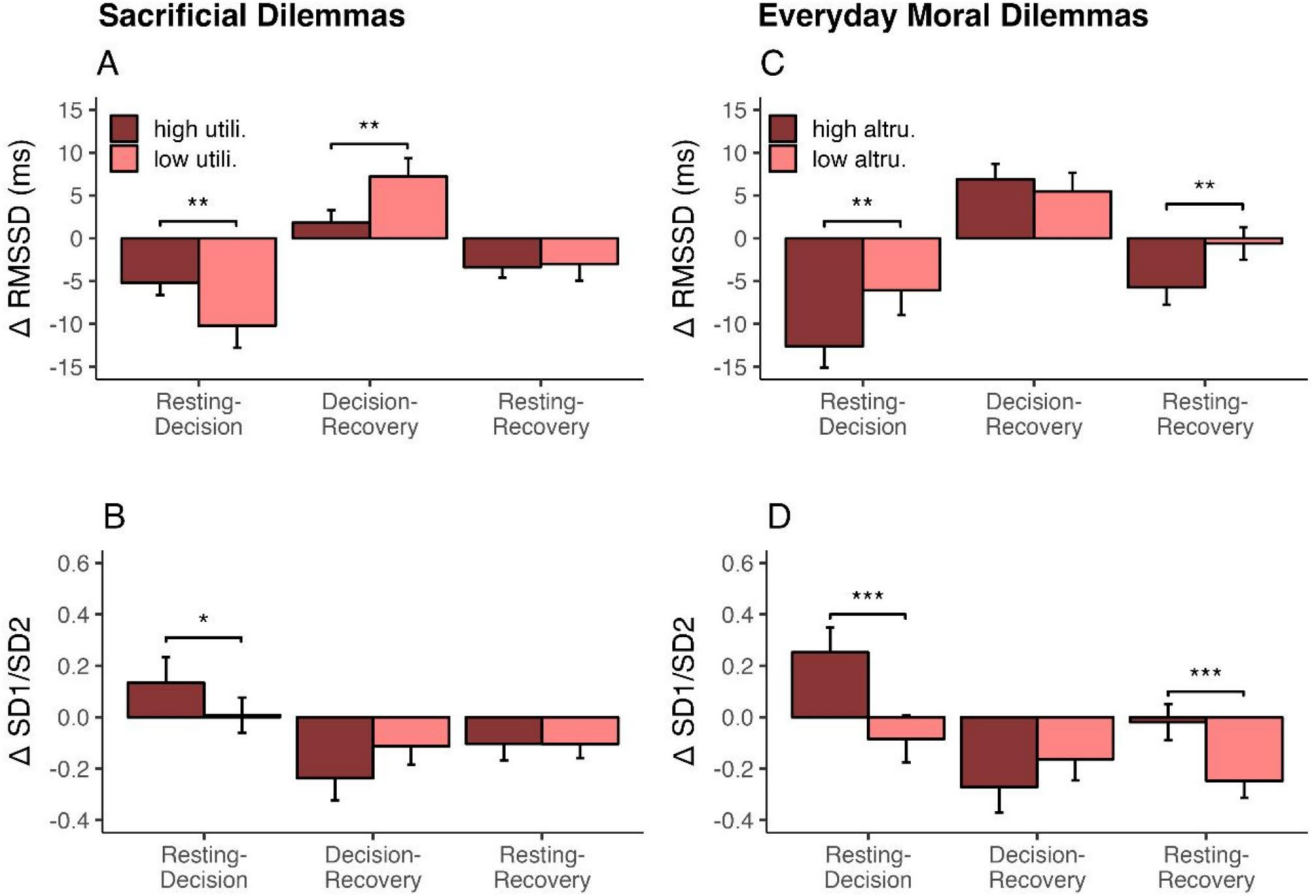
Differences in utilisation of self-regulatory capacity as indicated by HRV delta values. Group differences for women. Left: high utilitarian women in darker bars. Right: high altruistic women in darker bars. Delta values as adjusted means ± SE of HRV indices. Resting-Decision: difference (Δ) between resting and decision-making values; Decision-Recovery: Δ between decision-making and recovery values; Resting-Recovery: Δ between resting and recovery values. Asterisks indicate significant differences between the groups. (**A** and **C**) RMSSD, root mean square of successive differences; (**B** and **D**) SD1/SD2, short-term to long-term HRV. ***p < 0.001, ** *p* < 0.01, * *p* < 0.05.

The second study objective related to self-regulatory differences between decision-makers (low- versus high-utilitarian) and whether these could be predicted using HRV analysis. For the criteria of the classification low- vs. high-utilitarian, see the section *Data Analysis*. Of the 77 women, *n* = 46 were low-utilitarian and *n* = 31 were high-utilitarian decision-makers. Of the 35 men, *n* = 18 were low-utilitarian and *n* = 17 were high-utilitarian decision-makers. Results from generalised linear regression showed no significant differences between low- and high-utilitarian male decision-makers. In women, there were differences between low- and high-utilitarian decision-makers in all HRV domains. For RMSSD and HF, the ∆ resting - decision-making was negative in both groups and lower in the low-utilitarian group. Also, sympatho-vagal balance (LF/HF, SD1/SD2) was lower in the low-utilitarian group. The ∆ decision-making - recovery switched to positive values for the women with fewer utilitarian decisions, whereas highly utilitarian women only showed positive values for RMSSD. In the other indices, it remained negative. The ∆ resting - recovery showed no significant differences between the decision groups. Figure 2, panels A and B, shows differences in the utilisation of self-regulatory capacities in selected HRV indices. Detailed results for group differences in sacrificial decision-making indexed by HRV ∆ values are displayed in Table 1.

**Table 1:** Sacrificial moral decision-making: differences in utilisation of self-regulatory capacities as indicated by HRV delta values (Δ).

HRV indices	Utilitarian decisions	Resting - decision			Decision - recovery			Resting - recovery		
		*M ± SD*	*η*_*p*_^*2*^	*p*	*M ± SD*	*η*_*p*_^*2*^	*p*	*M ± SD*	*η*_*p*_^*2*^	*p*
Δ RMSSD (ms)	Low	-10.2 ± 17.3	0.02	< 0.01**	7.21 ± 14.3	0.04	< 0.01**	-3.00 ± 13.1	0.0002	0.80
	High	-5.19 ± 7.96			1.82 ± 8.04			-3.37 ± 6.74		
Δ HF pow (ms^2^)	Low	-259. ± 905.	0.02	< 0.01**	208. ± 731.	0.04	< 0.001***	-50.5 ± 719.	0.0002	0.83
	High	-38.3 ± 329.			-63.4 ± 330.			-67.2 ± 258.		
Δ LF/HF	Low	-0.83 ± 2.05	0.02	< 0.05*	0.26 ± 2.64	0.01	0.10	-0.57 ± 1.56	0.006	0.24
	High	0.09 ± 4.16			-0.33 ± 2.89			-0.24 ± 2.68		
Δ SD2 (ms)	Low	-12.5 ± 17.4	0.04	< 0.001***	4.64 ± 16.8	0.05	< 0.001***	-7.83 ± 14.8	0.00009	0.88
	High	-5.28 ± 12.5			-2.31 ± 11.3			-7.59 ± 8.43		
Δ SD2/SD1	Low	0.008 ± 0.45	0.01	< 0.05*	-0.11 ± 0.49	0.01	0.05	-0.10 ± 0.36	0.00009	0.98
	High	0.13 ± 0.55			-0.23 ± 0.47			-0.10 ± 0.35		
Δ DFA1	Low	-0.04 ± 0.20	0.004	0.31	-0.01 ± 0.20	0.0006	0.69	-0.04 ± 0.15	0.002	0.41
	High	-0.02 ± 0.17			-0.01 ± 0.16			-0.03 ± 0.14		

Sample of 77 women: *n* = 46 were low-utilitarian (≤ 50% utilitarian choices) and *n* = 31 were high-utilitarian. Significance Levels: ***p < 0.001, **p < 0.01, *p < 0.05.

### Everyday moral decision-making

Regarding the first study objective, cardiac vagal tone increased during decision-making in everyday dilemmas (women’s RMSSD by 33.31%, *p* < 0.001; men’s RMSSD by 26.68%, *p* < 0.01) and decreased during recovery (women’s RMSSD by -19.96%, *p* < 0.01; men’s RMSSD by -11.57%, *p* = 0.29), resembling a similar pattern as in sacrificial dilemmas. Complementary, DFA1 increased during decision-making by 10.64% in women (*p* < 0.05), but only slightly by 6% in men (*p* = 0.38). Again, it increased in men (6.36%, *p* = 0.33), ending in a significant difference between resting and recovery (12.37%, *p* < 0.05) for men, but not for women (7.51%, *p* = 0.14). Similar to sacrificial dilemmas, the differences between women and men did not reach significant levels. Sympatho-vagal balance increased continuously, reaching the highest levels during recovery (women’s LF/HF by 126.75%, *p* < 0.05; men’s by 124.22%, *p* < 0.05). Figure 1, panels C and D, shows the percentage changes from resting to everyday moral decision-making and recovery in selected HRV indices. Detailed results are summarised in the Supplementary Material (Table S3-S4).

Regarding the second study objective, HRV differences between decision-makers (low- versus high-altruistic) were assessed. For the classification criteria, see section *Data Analysis*. Similarly to sacrificial dilemmas, in the EMDM, *n* = 46 women were low-altruistic, and *n* = 31 were high-altruistic decision-makers. Of the 35 men, *n* = 23 were low-altruistic, and *n* = 12 were high-altruistic decision-makers. There were no differences between low- and high-altruistic male decision-makers, but for women in all HRV domains. In the ∆ resting - decision-making RMSSD was negative in both groups and significantly lower in the highly altruistic group. At the same time, sympatho-vagal balance (SD1/SD2, DFA1) was negative in low-altruistic but positive in high-altruistic women. In the ∆ decision-making - recovery, DFA1 was negative in high- and positive in low-altruistic women. The ∆ resting - recovery showed lower values for low-altruistic women in sympatho-vagal balance (SD1/SD2, DFA1) and higher values in vagal tone (RMSSD). Figure 2, panels C and D, shows differences in the utilisation of self-regulatory capacities in selected HRV indices. Detailed results are displayed in Table 2.

**Table 2:** Everyday moral decision-making: differences in utilisation of self-regulatory capacities as indicated by HRV delta values (Δ).

	Altruistic decisions	Resting - decision			Decision - recovery			Resting - recovery		
		*M ± SD*	*η*_*p*_^*2*^	*p*	*M ± SD*	*η*_*p*_^*2*^	*p*	*M ± SD*	*η*_*p*_^*2*^	*p*
Δ RMSSD (ms)	Low	-6.08 ± 19.3	0.03	< 0.01**	5.47 ± 14.6	0.002	0.41	-0.60 ± 12.8	0.04	< 0.01**
	High	-12.6 ± 13.6			6.90 ± 9.87			-5.73 ± 11.2		
Δ HF pow (ms^2^)	Low	-106 ± 1028	0.004	0.31	166. ± 917.	0.00002	0.90	59.2 ± 583.	0.008	0.15
	High	-236 ± 849			179. ± 613.			-57.2 ± 652.		
Δ SD2 (ms)	Low	-9.41 ± 18.6	0.004	0.33	3.23 ± 14.3	0.001	0.52	-6.18 ± 14.2	0.01	< 0.05*
	High	-12.0 ± 22.1			1.83 ± 18.8			-10.2 ± 15.1		
Δ SD2/SD1	Low	-0.08 ± 0.61	0.07	< 0.001***	-0.16 ± 0.55	0.009	0.14	-0.24 ± 0.44	0.06	< 0.001***
	High	0.25 ± 0.52			-0.27 ± 0.54			-0.01 ± 0.38		
Δ DFA1	Low	-0.09 ± 0.28	0.05	< 0.001***	0.01 ± 0.24	0.02	< 0.05*	-0.07 ± 0.17	0.02	< 0.05*
	High	0.03 ± 0.22			-0.05 ± 0.20			-0.01 ± 0.17		

^a^Sample of 77 women: *n* = 46 were low-altruistic (≤ 50% altruistic choices) and *n* = 31 were high-altruistic. Significance Levels: ***p < 0.001, **p < 0.01, *p < 0.05.

## Discussion

The scope of this study was to investigate self-regulation via HRV in different moral decision-making situations. To this end, the self-regulatory demands of two moral tasks were examined, and HRV analysis was used to differentiate concrete moral decision-making in the two tasks. Two main findings emerge from this study: first, HRV analysis is highly useful for revealing how self-regulatory demands are managed in the face of different moral contexts; second, HRV analysis is suitable for distinguishing between moral inclinations shaping concrete decision-making.

For self-regulatory demands, vmHRV was expected to increase during decision-making compared to rest and then decrease during recovery, in line with vagal tank theory^33^ and previous findings^37^. This replenishment effect was reflected in the current data: compared to rest, cardiac vagal control increases during decision-making and then decreases during recovery. This was found in both women and men, and in both sacrificial and everyday dilemmas. The replenishment effect can be interpreted as an adaptive self-regulatory process because an increase in vagal outflow provides the necessary resources to meet the demands of the task, for example, by enhancing concentration^33^. The observation of cardiac autonomic modulation during different phases of activation (i.e., resting, decision-making, recovery) tells us a lot about the nature of the task itself and the underlying adaptation mechanisms required to manage it. For example, a decrease in vmHRV in moral tasks would indicate an increased level of stress or cognitive load. It is interesting to note that, overall, this was not the case for either the sacrificial or the everyday dilemmas. In future research, it would be interesting to see which factors would lead to a depletion effect (vagal withdrawal during the task), for example, a higher degree of emotional involvement. The threshold is particularly interesting here: at what “moral burden” does the replenishment effect collapse or even change into a depletion of cardiac vagal control?

Our data provide unique insights into cardiac dynamics to different moral challenges. So far, the investigation of resting HRV dominates research, as it is a suitable index of trait-like vagal tone^38^. In the current study, we also analysed HRV during decision-making and recovery. This approach allows the assessment of state-related HRV. While conventional analysis of absolute HRV values provides general information about ANS modulation, the calculation of delta values provides additional dimensions about specific changes in HRV over time. They indicate whether HRV has increased (positive delta) or decreased (negative delta), thus revealing acute and subtle changes in ANS modulation in response to and recovery from a specific challenge. A sudden decrease in vmHRV can indicate a sudden stress response, whereas a rapid return to resting values signals an effective ability to recover/self-regulate. Additionally, delta values normalise for individual differences due to hormonal cycles, stress, training, illness or other factors, providing more precise insight into underlying mechanisms and avoiding confounding factors. The calculation of the delta values was of methodological value for the second study objective, namely to identify different patterns in utilising self-regulatory capacities between concrete moral decisions.

Another methodological strength of this study was the stratification of the sample by sex, which reduced potential confounding effects and minimised the risk that effects would not be reliably captured if sex was treated as an exploratory variable or in a secondary analysis^39^. Indeed, the analysis of HRV to discriminate concrete moral decisions in both dilemma tasks showed effects only for women.

During decision-making in sacrificial dilemmas, a lower vmHRV was apparent in low-utilitarian (i.e., deontological) women Lower delta values suggest that low-utilitarian individuals react more strongly to the decision-making process itself. A lower vmHRV could indicate various regulatory difficulties, for example, a stronger cardiac vagal stress response, difficulties in the effective regulation of emotions, insufficient capacity to maintain cognitive control, or overall lower ability to adapt to moral demands. Lower vmHRV in low-utilitarian women might be interpreted according to dual process theory^4^: utilitarian decisions seem to be mainly based on cognitive processes and deliberation. Difficulties in recruiting cognitive processes interfere with utilitarian decision-making. This study showed that a vagal withdrawal during sacrificial decision-making predicted fewer utilitarian decisions in women. Noteworthy, indices of sympatho-vagal balance were higher during decision-making in the high-utilitarian female group. This indicates increased sympathetic activity in this group. The sympathetic overdrive may reflect a trade-off in which cognitive effort required for utilitarian reasoning suppresses emotional responses. Furthermore, our data reveal that recovery patterns differed between high- and low-utilitarian female decision-makers: low-utilitarian individuals showed increased vmHRV, indicating faster recovery, whereas lower vagal tone was related to a high proportion of utilitarian decisions. This suggests that decisions driven more by deontological reasoning may not require the same level of cognitive effort or sympathetic engagement as utilitarian decision-making, facilitating faster physiological recovery. An alternative explanation may be that decision-makers with a low utilitarian preference have greater autonomic flexibility, allowing them to return quickly to the resting state after decision-making. Remarkably, the high-utilitarian decision-makers show smaller variations (decreases and increases) of HRV across the measurement phases compared to low-utilitarian decision-makers. Stein et al.^40^ argue that too little variation may indicate an inadequate function of the self-regulatory control systems at various levels (e.g., ANS dysregulation, difficulties in neurovisceral integration). It may be that high-utilitarian decision-making is associated with physiological costs or maladaptive strategies that could have long-term effects on overall health. Also, in light of findings by Park et al.^20^ and Rosas et al.^22^, who found that lower resting vmHRV was associated with higher utilitarian judgement, further research should investigate whether lower HRV fluctuations represent maladaptive compensatory mechanisms in moral decision-making.

In sum, the current data suggest that high- and low-utilitarian women have different patterns of cardiac vagal control. However, the exact mechanisms by which self-regulation, as measured by vmHRV, predicts actual moral decision-making remain unclear. Further research is needed to understand how exactly HRV is related to (moral) decision-making. Possible approaches for this are intervention or biofeedback studies. For example, Bornemann et al.^41^ showed that participants could intentionally increase their HF-HRV in a biofeedback task and that this ability predicted individual differences in prosocial behaviour. De Couck et al.^42^ showed that skewed patterns (exhalation longer than inhalation) of vagal breathing increased HRV and improved decision-making in a multiple-choice task. These examples suggest that HRV may influence decision-making, but studies are scarce and vary widely in methodology, so no general conclusions can be drawn at present. In particular, the methodological aspect of moral dilemmas needs to be considered. The nature of dilemmas is that there is no right or wrong answer. Accordingly, moral performance cannot be quantified, and it is difficult to reconcile decision performance with regulatory effort^22^. Future research should therefore focus particularly on the methodological possibilities of capturing context-specific autonomic regulation to determine how autonomic responses may vary depending on the nature of the moral decision-making task.

Advancing the debate, this study investigated cardiac autonomic modulation in everyday moral decision-making. We found different cardiac vagal responses, depending on the altruistic level of the person, but only for women. Highly altruistic women showed a lower vmHRV, which could indicate a reduced ability of the ANS to adapt to moral demands. At the same time, the sympathetic dominance in the sympatho-vagal balance indicates an increased stress response or increased stress in the highly altruistic group. The same was reflected in the resting - recovery comparison, which showed lower vmHRV and, at the same time, a sympathetic dominance in highly altruistic individuals compared to low-altruistic individuals. Although there is evidence that psychological stress affects everyday moral decision-making, the results are inconsistent. For example, Singer et al.^43^ demonstrated a tendency for people to act more altruistically under acute stress, particularly in women, while Starcke et al.^29^ found a positive correlation between cortisol stress responses and egoistic decision-making in a gender-mixed sample. A meta-analysis of 23 studies and 2197 participants found no moderating effects of stressor type, gender, or time between stressor and task^44^. The authors conclude that there currently is no clear answer to the question of whether stress is associated with increased or decreased prosociality. Our data show that altruistic decisions in everyday moral dilemmas under laboratory conditions are associated with reduced vagal outflow and increased sympathetic dominance in women, which may indicate a cardiac stress response. Further research is needed to clarify this nexus.

The lack of effects in men across both moral dilemma tasks may stem from biological factors, differences in decision-making strategies, or methodological aspects. Biologically, women have a higher vmHRV than men^30–32^, especially when confronted with emotional stimuli^45^, which may make HRV-related effects on emotional and cognitive processes more detectable in women. Regarding moral decision-making, some authors attribute sex differences to stronger affective responses to harm in women compared to men^46^. If HRV is closely linked to affective regulation and women rely more on emotional processing when making moral decisions, this could explain why effects are more likely to be observed in women. However, due to the diverse methodological approaches employed—including variations in study design, sample characteristics, decision paradigms, and statistical control—conclusions on sex-specific differences remain equivocal. Future research should explore these aspects to ensure a more accurate assessment of sex differences in HRV and moral decision-making.

The analysis of HRV has untapped potential in sensitively detecting differences between concrete decisions in both sacrificial and everyday moral decision-making. This potential needs to be further exploited, also in other fields of decision-making. For example, Alacreu-Crespo et al.^47^ demonstrated that the intuitive decision-making style (i.e., decisions based on subjective impressions or gut feelings) predicted HF-HRV and the avoidant style predicted less LF-HRV. Prell et al.^37^ found that women and men who made disadvantageous decisions under ambiguity had higher vmHRV than advantageous decision-makers and that vmHRV was lower in high-risk men than in women.

Some limitations need to be considered. The men’s sample was smaller than the women’s, so there was a gender imbalance. The results should be replicated with larger samples. Also, we are aware that sex-stratified data must be treated with caution because subgroup analyses of main effects have an increased risk of type II errors or false-negative results^48^. Nevertheless, HRV data can differ greatly between the sexes, and it seems that most HRV studies have neglected these differences by not adequately controlling for them, which is why we decided to opt for this approach and additionally calculated the delta values. For the HRV measurements, we did not directly measure respiratory rate. Changes in respiratory rate during the measurements may have contributed to the observed changes in HF-HRV. Although the treatment of respiratory rate is controversial, we followed the recommendations of Laborde et al.^49^ and did not routinely correct HRV for spontaneous breathing.

To conclude, in this study, we show that HRV analysis assesses dynamic changes related to different situational demands, and detects individual differences in moral decision-making in both extreme and everyday situations. Our data suggest that lower vmHRV is associated with fewer utilitarian decisions in sacrificial dilemmas, possibly indicating a disutilisation of self-regulatory capacities. Nevertheless, women with low utilitarianism show faster cardiac vagal recovery compared to high utilitarian women, possibly indicating a trade-off between cognitive and emotional responses. In everyday moral dilemmas, lower vmHRV is associated with higher levels of altruism in women, possibly indicating a disutilisation of self-regulatory capacities. The results suggest differences between women and men that should be investigated in the future. Implications of the current results are far-reaching: HRV analysis provides insight into subtle changes, such as cognitive effort or emotional states^50^. This allows for the development of targeted, individualised interventions to support the decision-making process and the simultaneous assessment of the interventions’ effectiveness using an objective marker. The increasing availability and reliability of small and lightweight devices to record HRV makes it a very powerful tool that provides continuous insight into an individual’s ability to adapt/self-regulate.

## Methods

The ethics committee of the University of Duisburg-Essen approved the study (ID: 2103APPR4635). All participants were informed of the study procedures, data collection, and anonymisation of all personal data, and provided informed consent before participation. The study methods were carried out following relevant guidelines, and the study was conducted in accordance with the Declaration of Helsinki.

### Participants

Using G*Power^51^, a priori power analysis indicated a sample size of *N* = 84 to be sufficient to detect an effect of *η*_*p*_^*2*^ = 0.025 (effect size from Forte et al.) with a statistical power of 1-β = 0.9 and α = 0.05 in a repeated measures ANOVA model (within-between interaction, three measurements, two groups). A separate power analysis for the regression analysis (one predictor) indicated that a sample size of *N* = 73 would be adequate to detect an effect of *η*_*p*_^*2*^ = 0.06 (effect size from Park et al.^53^) with statistical power of 1-β = 0.9 and α = 0.05. To mitigate the risk of underpowering in HRV studies^54^ and potential dropouts, 117 participants with different educational backgrounds—students, graduates, apprentices, and employees—in the age range from 18 to 40 years were recruited. Participants with medical or psychiatric diagnoses were excluded, as were those who reported using cardioactive medications, or antihypertensive beta-blockers. Heart rate recordings with more than 3% ectopic beats were not included in the data analysis.

### Moral decision-making

*Sacrificial moral decision-making*: Twenty moral dilemma vignettes were used, which had previously been employed in numerous studies in moral psychology, involving a conflict between utilitarian and deontological moral foundations^5,8^. Across all vignettes, the average distribution of words and characters was balanced and the number of people to be saved was carefully matched (see recommendations^55^). The utilitarian versus deontological inclination was queried with a dichotomous “yes” or “no” question, with the “yes” response indicating the utilitarian preference. The percentage of utilitarian decisions was calculated, in line with previous research^9,10^.

*Everyday moral decision-making*: The Everyday Moral Decision-Making Task (EMDM^29^) was used to assess conflicts between altruistic and egoistic moral foundations. The EMDM is a collection of 20 moral vignettes designed to be as close to everyday life as possible. An example is the unobserved, accidental scratching of a parked car. When asked whether one would report the incident, the answer “yes” is the altruistic alternative, while the answer “no” is the egoistic one. The percentage of altruistic decisions was calculated, in line with previous research^9^.

### Heart rate variability

The Bittium Faros™ 180 (Bittium, Oulu, Finland) was used to record a one-lead electrocardiogram (ECG) using the 3-electrode system at a sampling rate of 1000 Hz. In addition to the mean heart rate (HR), the HRV was analysed in time, frequency, and non-linear domains. In the time domain, the root mean square of successive differences (RMSSD) was used as an index for vagal tone^38^. In the frequency domain, the high frequency (HF) power was selected, which depends mainly on vagal activity, being synchronous with respiratory sinus arrhythmia^13^. Using Fast Fourier Transformation, the HF components were expressed in absolute units (ms^2^/Hz). Additionally, the low-to-high frequency (LF/HF) power ratio was assessed for the sacrificial dilemmas only, reflecting sympatho-vagal balance or sympathetic modulation^13^. As a complementary non-linear HRV indicator, the standard deviation of continuous long-term RR interval variability (SD2) was used. SD2 can be interpreted as a sign of both vagal and sympathetic tone^56^ and correlates with LF HRV^57^. In addition, the SD1/SD2 ratio was used to assess the relationship between variation in the short interval (SD1) and variation in the long interval (SD2), and thus the sympatho-vagal balance for both sacrificial and everyday dilemmas. Furthermore, detrended fluctuation analysis (DFA1) was considered to support the findings regarding the sympatho-vagal balance, as DFA1 increases when vagal tone decreases^58^.

### Experimental procedure

Participants were instructed to refrain from strenuous physical activity, alcohol, or other drugs for at least 24 h and stimulant drinks (e.g., coffee, tea, energy drinks) or food for at least two hours prior to measurement. From June to October, a maximum of two participants were measured simultaneously between 9 am and 1 pm in an experimental room with controlled lighting (4000 K, 900 –2400 lm) and temperature (20.21 ± 0.75 °C). After the application of disposable Kendall™ H66LG Ag/AgCl liquid gel electrodes, participants were seated on a non-moving chair in front of a computer. In line with HRV measurement standards^13,59^, participants maintained the same body position throughout the data collection: seated body position, knees bent at a 90° angle, both feet flat on the floor. After a one-minute acclimatisation period, a 10-minute resting HRV recording began. This was followed by four different decision tasks, including the two moral dilemma tasks. The results for decisions under ambiguity (IGT^60^) and risk (GDT^61^) are reported elsewhere. One dilemma at a time was presented in full-screen mode in the participant’s native language (i.e., German) in random order in Courier 36 pt font^55^. To reduce switching costs and interference, there was a one-minute break between each task^62^. The tasks were followed by a 10-minute recovery phase.

### Data analysis

The Kubios HRV Premium Software (ver. 3.5, Kubios Oy, Kuopio, Finland) was used to process the ECG traces. The automatic beat correction filters were set to “very low”. The ECG traces were visually inspected by the same trained operator. The HRV analysis windows were aligned with the processing time of the moral decision tasks: for the sacrificial dilemmas, the analysis window between resting, decision-making, and recovery was 10 min; for the EMDM it was 4 min each. The processing time of the EMDM, and thus the HRV analysis windows were below the 5-minute standard for short-term measurements^13^, but recent studies on the validity of ultra-short HRV analysis have shown that recordings longer than 2 min appear to be reliable for the accuracy of HRV analysis^63–65^.

Statistical analyses were performed using R statistical software (R Core Team, 2025). The sample was stratified by sex to take into account that HRV varies greatly depending on sex (see Introduction). All data were tested for normality (Q-Q plots and Shapiro-Wilk test), variance homogeneity (Levene test), and sphericity (Bartlett test).

To assess cardiac autonomic self-regulation across different measurement time points (i.e., resting, decision-making, and recovery), HRV values were normalised on an individual basis. For this purpose, HRV values during decision-making and recovery were calculated as percentage changes from the resting value, which served as a 100% reference value (i.e., decision-making values, and recovery values divided by resting values). To evaluate the HRV changes between the measurement times, a repeated measures ANOVA with Tukey HSD post hoc analysis and Tukey p-value adjustment was conducted. Effect sizes were calculated with eta-squared (*η*^2^).

To assess differences in cardiac autonomic modulation not only across different measurement times but also between the concrete moral decisions, the participants were allocated into two groups. Based on the percentage of utilitarian responses, participants were assigned to either a high- or low-utilitarian group^66^. Participants who chose ≤ 50% of the “yes” response were in the low-utilitarian (i.e., deontological) group, and those who chose > 50% of the “yes” response belonged to the high-utilitarian group. For the EMDM, participants were similarly categorised into low-altruistic (i.e., egoistic) and high-altruistic groups. The HRV data was normalised for each participant by calculating HRV delta values (∆). Delta values were calculated by (1) resting values minus decision-making values, (2) decision-making minus recovery values, and (3) resting minus recovery values. The Δ thus indicates the difference between resting values and decision-making values, as well as the quality of recovery after decision-making (i.e., whether after decision-making values immediately returned to resting level). Group differences in moral decision-making were analysed by generalised linear regression model with HRV indices as predictors and decision-making (e.g., group classification as low- or high-utilitarian/altruistic) as the dependent variable. For HRV indices that did not follow a normal distribution, a gamma distribution with a log-link function was applied to appropriately model skewed HRV data. Effect sizes were calculated with partial eta-squared (*η*_*p*_^2^). In all testing, the significance level was set at α = 0.05 with a 95% confidence level.

## Electronic supplementary material

Below is the link to the electronic supplementary material.

Supplementary Material 1

## Data Availability

The datasets generated during and/or analysed during the current study are available from the corresponding author on reasonable request.
