# Supplementary material for: Cardiac vagal modulation predicts decision-making in sacrificial and everyday moral dilemmas
Source: Sci Rep. 2025 Apr 15;15:13029. doi: 10.1038/s41598-025-96475-9 (PMC12000318; doi:10.1038/s41598-025-96475-9)
Supplement: Supplementary file 1 — Supplementary Material 1 [file 41598_2025_96475_MOESM1_ESM.pdf]

Supplementary Materials

Cardiac vagal modulation predicts decision-making in sacrificial and everyday moral dilemmas

Rebecca Prell[a] *, Martina Anna Maggioni[b,c] and Katrin Starcke[d,e]

[a] General Psychology: Cognition, University of Duisburg-Essen, Germany; [b] Charité—Universitätsmedizin Berlin, Institute of Physiology, Center for Space Medicine and Extreme Environments, Berlin, Germany; [c] Department of Biomedical Sciences for Health, Università Degli Studi di Milano, Milan, Italy; [d] SRH Berlin School of Applied Sciences, Berlin, Germany; [e] Berlin Institute of Biomusicology and Empirical Research, Berlin, Germany

*Correspondence:

Rebecca Prell, M. Sc.
University of Duisburg-Essen
Forsthausweg 2
47057 Duisburg
Tel: +49 30 616548-0
rebecca.prell@stud.uni-due.de

Rebecca Prell ORCiD: https://orcid.org/0000-0003-1682-7590

Katrin Starcke ORCiD: https://orcid.org/0000-0001-7897-7418

Martina Anna Maggioni ORCiD: https://orcid.org/0000-0002-6319-8566

Table S1. Sacrificial Moral Decision-Making: Main Effects of Repeated Measures ANOVA for Percentage Change in HRV Across the Measurement Times.

	Women n = 77			Men n = 35		
HRV Indices	F [a]	$\eta 2$	p	F [b]	$\eta 2$	p
**Time Domain**						
% RMSSD	17.25	0.13	< 0.001***	4.18	0.07	0.01*
**Frequency Domain**						
% HF power	8.06	0.06	< 0.001***	2.07	0.03	0.13
% LF/HF	14.15	0.11	< 0.001***	6.17	0.10	0.002**
**Non-linear**						
% SD2/SD1	3.93	0.03	0.02*	4.92	0.08	0.009**
% DFA1	3.63	0.03	0.02*	7.04	0.12	0.001**

[a] df = 2, 228; [b] df = 2, 102. Significance Levels: ***p < 0.001, **p < 0.01, *p < 0.05.

Table S2. Sacrificial Moral Decision-Making: Tukey HSD Multiple Comparisons of Means Post hoc Analysis of Repeated Measures ANOVA for Percentage Change in HRV Across the Measurement Times.

	Women n = 77					Men n = 35				
			95% CI					95% CI		
HRV Indices	*Mean difference* [a]	*SE*	*Lower bound*	*Upper bound*	*p*	*Mean difference* [b]	*SE*	*Lower bound*	*Upper bound*	*p*
**Time domain**										
% RMSSD										
Resting x Decision	29.11	4.96	17.42	40.81	< 0.001***	19.40	6.80	3.23	35.57	0.01*
Decision x Recovery	-14.11	4.96	-25.81	-2.42	0.01*	-6.84	6.80	-23.01	9.33	0.57
Resting x Recovery	14.99	4.96	3.30	26.69	0.007**	12.56	6.80	-3.60	28.73	0.15
**Frequency domain**										
% HF power										
Resting x Decision	54.08	13.80	21.60	86.56	< 0.001***	39.99	19.60	-6.67	86.65	0.10
Decision x Recovery	-17.04	13.80	-49.51	15.43	0.43	-19.35	19.60	-66.01	27.31	0.58
Resting x Recovery	37.04	13.80	4.56	69.52	0.02*	20.63	19.60	-26.02	67.30	0.54
% LF/HF ratio										
Resting x Decision	70.97	13.50	39.15	102.79	< 0.001***	55.37	21.40	4.44	106.29	0.02*
Decision x Recovery	-26.36	13.50	-58.18	5.45	0.12	16.42	21.40	-34.49	67.34	0.72
Resting x Recovery	44.60	13.50	12.78	76.42	0.003**	71.79	21.40	20.87	122.71	0.003**
**Non-linear**										
% SD2/SD1 ratio										
Resting x Decision	-0.45	2.44	-6.21	5.30	0.98	0.76	3.37	-7.24	8.77	0.97
Decision x Recovery	6.14	2.44	0.38	11.90	0.03*	8.74	3.37	0.73	16.75	0.02*
Resting x Recovery	5.69	2.44	-0.06	11.45	0.05	9.51	3.37	1.49	17.52	0.01*
% DFA1										
Resting x Decision	6.41	2.67	0.11	12.71	0.04*	3.38	2.93	-3.57	10.34	0.48
Decision x Recovery	-0.39	2.67	-6.68	5.90	0.98	7.35	2.93	0.39	14.31	0.03*
Resting x Recovery	6.02	2.67	-0.27	12.32	0.06	10.74	2.93	3.77	17.70	0.001**

[a] df = 228; [b] df = 102. Multiple post hoc comparisons with Tukey correction to adjust p-values were performed to compare the mean difference between Resting, Decision-Making, and Recovery. Standard Error (SE); Confidence Interval (CI). Significance Levels: ***p <0.001, **p < 0.01, *p < 0.05.

Table S3. Everyday Moral Decision-Making: Main Effects of Repeated Measures ANOVA for Percentage Change in HRV Across the Measurement Times.

	Women n = 77			Men n = 35		
HRV Indices	F^{a}	$\eta2$	*p*	F^{b}	$\eta2$	*p*
**Time Domain**						
% RMSSD	15.98	0.12	< 0.001***	6.03	0.10	0.003**
**Frequency Domain**						
% HF power	9.47	0.07	< 0.001***	6.29	0.10	0.002**
% LF/HF	8.71	0.07	< 0.001***	3.46	0.06	0.03*
**Non-linear**						
% SD2/SD1	4.30	0.03	0.01*	5.03	0.08	0.008**
% DFA1	3.70	0.03	0.02*	3.76	0.06	0.02*

[a] df = 2, 228; [b] df = 2, 102. Significance Levels: ***$p < 0.001$, **$p < 0.01$, *$p < 0.05$.

Table S4. Everyday Moral Decision-Making: Tukey HSD Multiple Comparisons of Means Post hoc Analysis of Repeated Measures ANOVA for Percentage Change in HRV Across the Measurement Times.

	Women n = 77					Men n = 35				
			95% CI					95% CI		
HRV Indices	*Mean difference* [a]	*SE*	*Lower bound*	*Upper bound*	*p*	*Mean difference* [b]	*SE*	*Lower bound*	*Upper bound*	*p*
**Time domain**										
% RMSSD										
Resting x Decision	33.31	5.93	19.32	47.31	< 0.001***	26.68	7.71	8.36	45.01	0.002**
Decision x Recovery	-19.96	5.93	-33.96	-5.97	0.002**	-11.57	7.71	-29.89	6.75	0.29
Resting x Recovery	13.35	5.93	-0.64	27.34	0.06	15.11	7.71	-3.21	33.44	0.12
**Frequency domain**										
% HF power										
Resting x Decision	56.94	13.10	26.06	87.83	< 0.001***	76.82	22.30	23.79	129.84	0.002**
Decision x Recovery	-30.10	13.10	-60.98	0.78	0.05	-54.83	22.30	-107.85	-1.81	0.04*
Resting x Recovery	26.84	13.10	-4.03	57.73	0.10	21.98	22.30	-31.03	75.00	0.58
% LF/HF ratio										
Resting x Decision	88.31	31.10	53.32	200.19	< 0.001***	73.36	47.50	-39.55	186.27	0.27
Decision x Recovery	-38.43	31.10	-111.87	34.99	0.43	50.86	47.50	-62.05	163.77	0.53
Resting x Recovery	126.75	31.10	14.88	161.75	0.01*	124.22	47.50	11.30	237.14	0.02*
**Non-linear**										
% SD2/SD1 ratio										
Resting x Decision	0.48	3.18	-7.02	8.00	0.98	3.87	5.15	-8.37	16.13	0.73
Decision x Recovery	7.83	3.18	0.32	15.34	0.03*	11.81	5.15	-0.43	24.06	0.06
Resting x Recovery	8.32	3.18	0.81	15.83	0.02*	15.69	5.15	3.44	27.94	0.008**
% DFA1										
Resting x Decision	10.64	4.02	1.16	20.12	0.02*	6.00	4.51	-4.71	16.72	0.38
Decision x Recovery	-3.13	4.02	-12.60	6.34	0.71	6.36	4.51	-4.35	17.08	0.33
Resting x Recovery	7.51	4.02	-1.96	16.99	0.14	12.37	4.51	1.64	23.09	0.01*

[a] df = 228; [b] df = 102. Multiple post hoc comparisons with Tukey correction to adjust p-values were performed to compare the mean difference between Resting, Decision-Making, and Recovery. Standard Error (SE); Confidence Interval (CI). Significance Levels: ***p <0.001, **p < 0.01, *p < 0.05.
